# Immunotherapy in Gastrointestinal Cancers

**DOI:** 10.1155/2017/4346576

**Published:** 2017-07-03

**Authors:** Letizia Procaccio, Marta Schirripa, Matteo Fassan, Loredana Vecchione, Francesca Bergamo, Alessandra Anna Prete, Rossana Intini, Chiara Manai, Vincenzo Dadduzio, Alice Boscolo, Vittorina Zagonel, Sara Lonardi

**Affiliations:** ^1^Division of Medical Oncology 1, Istituto Oncologico Veneto, IRCCS, Padova, Italy; ^2^Department of Surgery, Oncology and Gastroenterology, University of Padova, Padova, Italy; ^3^Department of Medicine, Surgical Pathology & Cytopathology Unit, University of Padova, Padova, Italy; ^4^Division of Molecular Carcinogenesis, Cancer Genomics Center Netherlands, The Netherlands Cancer Institute, Amsterdam, Netherlands; ^5^Department of Radiological, Oncological and Pathological Sciences, Policlinico Umberto I University Hospital, Rome, Italy; ^6^Department of Medical Oncology, Fondazione Policlinico Universitario Agostino Gemelli, Università Cattolica del Sacro Cuore, Rome, Italy

## Abstract

Gastrointestinal cancers represent a major public health problem worldwide. Immunotherapeutic strategies are currently under investigation in this setting and preliminary results of ongoing trials adopting checkpoint inhibitors are striking. Indeed, although a poor immunogenicity for GI has been reported, a strong biological rationale supports the development of immunotherapy in this field. The clinical and translational research on immunotherapy for the treatment of GI cancers started firstly with the identification of immune-related mechanisms possibly relevant to GI tumours and secondly with the development of immunotherapy-based agents in clinical trials. In the present review a general overview is firstly provided followed by a focus on major findings on gastric, colorectal, and hepatocellular carcinomas. Finally, pathological and molecular perspectives are provided since many efforts are ongoing in order to identify possible predictive biomarkers and to improve patients' selection. Many issues are still unsolved in this field; however, we strongly believe that immunotherapy might positively affect the natural history of a subgroup of GI cancer patients improving outcome and the overall quality of life.

## 1. Gastrointestinal Cancers: Where Do We Stand?

Gastrointestinal (GI) cancers, including colorectal cancer (CRC), gastric cancer (GC), pancreatic cancer, and cancers of the liver (HCC) and of the biliary tract, are among the most frequent malignancies diagnosed annually in Europe and represent a major public health problem worldwide [1].

Although early-stage GI cancers are amenable to surgical resection with curative-intent, the overall 5-year relapse rate remains high. As a matter of fact, the addition of neoadjuvant or adjuvant chemotherapy and radiation therapy, when indicated, only modestly improves the overall long-term survival. Unfortunately, a large proportion of patients present with unresectable disease at the time of diagnosis: approximately 25% of GI cancers are diagnosed at advanced stage, whereas another 25 to 50% of patients will develop metastases during the course of the disease [2, 3]. In the last decade, meaningful improvement in the prognosis of patients with metastatic GI cancers derived from the development of new intensive and/or tailored therapies, which incorporated cytotoxic drugs and targeted therapies (cetuximab, panitumumab, bevacizumab, aflibercept, and regorafenib for mCRC; trastuzumab and ramucirumab for mGC; and sorafenib for HCC), and from the integration of medical treatments with more and more effective locoregional and surgical approaches [4]. Despite these advances, GI cancers are still a leading cause of cancer death [4]; thus, it is imperative to develop novel therapeutic approaches for patients affected by those cancers.

In recent years, we assisted in a paradigmatic shift in the treatment of both solid tumours, such as melanoma, non-small cell lung cancer, and genitourinary cancers, as well as hematologic malignancies, thanks to the striking results with long lasting responses and increased overall survival (OS) obtained with immunotherapy-based agents [5–7]. In parallel, the clinical and translational research on immunotherapy for the treatment of GI cancers started firstly with the identification of immune-related mechanisms possibly relevant to GI tumours and secondly with the development of immunotherapy-based agents in clinical trials.

Undoubtedly, the progress made towards the development of effective antitumour immunotherapies for GI cancers has been relatively slow: the first practice changing clinical data came out only in 2015 and the most part of immunotherapies are still in early phase clinical testing. The main reason for having GI cancers as a kind of Cinderella in the landscape of tumoural immunotherapy resides in the lack of their effector T cell responses and in the their well-known poor immunogenicity [8]. Immunotherapy against cancer has been assumed to be beneficial mainly in tumours with high immunogenicity by nature [9]. However, some approaches to circumvent immunosuppression including programmed death-1 (PD-1)/programmed death ligand-1 (PD-L1) blockade were successful to achieve significant response, also in cancers that hardly retain immunogenic nature [10].

This article highlights the state of the art of immunotherapy in GI deepening recent scientific evidence regarding anti-PD-1/PDL-1 and anti-CTLA4 monoclonal antibodies, peptide based vaccine, DNA based vaccine, and pulsed dendritic cells (DC), also outlining current clinical trials and finally suggesting areas for future research.

## 2. The Rationale for Immunotherapy in GI Cancers

Accumulating evidences indicate that a dynamic cross-talk between tumours and the immune system can regulate tumour growth and metastasis [10]. The increased understanding of the biochemical nature of tumour antigens and of the molecular mechanisms responsible for innate and adaptive immune cell activation has revolutionized the fields of tumour immunology and immunotherapy.

The first notion of a role of immunity in cancer was postulated in 1909 by Ehrlich, speculating that the immune system could repress the growth of carcinomas recognising tumour cells as foreign. About 50 years later, the theory of tumour immune surveillance was proposed by Burnet [11]. However, this theory has been recently completed with the identification of the so-called immunoediting proposed by Schreiber et al. The immunoediting progresses through 3 main phases: (1) the elimination phase (or immunosurveillance), when the innate and adaptive immune cells remove the proliferating cells, thus protecting the host against cancer; (2) the equilibrium phase, when the tumour growth and the immunosurveillance enter into a dynamic balance; in this genetically instable phase, the increase of mutational load and the emergence of resistant clones among tumour cells lead to (3) the escape phase; at this point, tumour variants are able to avoid immune-mediated destruction and speed up tumour progression and clinical expression [12, 13].

A role for the immunoediting in gastroenteropancreatic tumour pathogenesis was suggested since the first observations that T cells infiltration was linked to a more favorable outcome in pancreatic cancer, CRC, and GC [13, 14]. The following studies regarding the molecular basis and regulation of immunoediting have identified the tumour cells, the tumour microenvironment, and the immune system as the key players of a complex network [15]. Defining the relationships between these key players has been critical in facilitating the development of successful immunotherapies.

(A)* Tumour cells* have developed several mechanisms that directly or indirectly block the activity of effector antitumour CD4+ and CD8+ T cells dampening local tumour-infiltrating immune responses [16, 17]. Examples include (1) the secretion of soluble immunosuppressive factors (TGF-beta, IL-10, VEGF, and indoleamine 2,3 dehydrogenase) [18, 19]; (2) the activation of negative costimulatory signals in the tumour microenvironment such as PD-L1 [20, 21]; (3) tumour-induced impairment of the antigen presentation machinery due to the accumulation of point mutations in the cell surface not recognised by cytotoxic T cells [22]; and finally (4) the downregulation of the major histocompatibility complex (MHC) class I expression which plays a crucial role in tumour antigens presentation to T cells [22].

(B) Mechanisms explaining the* tumour microenvironment* role in immunoediting are best illustrated in studies on human and mouse pancreatic cancer models, since desmoplasia is the pathologic hallmark of pancreatic cancer [23, 24]. This inflammatory environment consists of regulatory immune cells, extracellular matrix proteins, and all the above fibroblasts (cancer-associated fibroblasts, CAFs) [25]. These stroma players in turn secrete tumour-promoting factors that contribute to tumour invasion and neoangiogenesis [26, 27]. Interestingly, CAFs have a critical role in CRC immunosuppression [28]: their activity in* RAS* mutant tumours overcome effector T cells signalling leading to tumour progression thanks to the activation of epithelial mesenchymal transition and TGF-beta/SMAD signalling [28]. Actually, high levels of CAFs markers correlated with poor prognosis in CRC [29].

(C)* The immune system* plays a critical role in immunoediting thanks to the involvement of several innate and adaptive effectors such as myeloid-derived suppressor cells (MDSCs), mast cells, tumour associated macrophages (TAMs), mesenchymal stem cells (MSCs), CD4+/CD25+ regulatory T cells (Tregs), and DCs [30]. By modulating the tumour microenvironment through the secretion of selected chemokines, cancer cells can actively prevent the induction of antitumour immunity through the differentiation, expansion, and/or recruitment of Treg [30, 31]. It has been reported that a low percentage of Tregs in the circulation 1 year after resection of pancreatic cancer correlates with improved survival [32, 33]. In addition, DCs are critically important for the generation and the maintenance of a specific adaptive antitumour immune response [33, 34]. Data from many laboratories obtained during past few years indicate that defects in DCs are among the main factors responsible for tumour escape [35].

Among immunosuppressive mechanisms the immune checkpoint modulation mediated by cytotoxic T-lymphocyte-associated antigen 4 (CTLA-4) and Programmed Death-1 (PD-1) plays a crucial role. In the normal host setting, immune checkpoint molecules modulate the T-cells response to antigens by either upregulating costimulatory pathways or downregulating coinhibitory pathways of immune signalling. CTLA-4 is an inhibitory receptor expressed by T cells. It can bind to CD80 or CD86 on DCs and inhibit their capabilities to activate T cells. CTLA-4 plays a critical role in the initial phase of immune response. PD-1 is a cell surface coinhibitory receptor that downregulates T cell activity in peripheral tissues during inflammation, thus preventing increased collateral tissue damage during an immune response and the development of autoimmunity. PD-1 is widely expressed on T cells, B cells, monocytes, and natural killer cells and plays a critical role in subsequent phases of immune responses compared to CTLA-4. It has two known ligands, PD-L1 and PD-L2, which are both upregulated during an inflammatory response. Tumour cells of various malignancies have been shown to upregulate PD-L1 as a mechanism that dampens the local T cell response by decreasing cytokine production and T cell proliferation. In GI malignancies, PD-L1 upregulation has been demonstrated to occur in pancreatic, GC, and CRC [34, 35], thus correlating with poor prognosis [35].

Moving from such complex background, immunotherapeutic strategies in GI cancers have been developed and are described in the following paragraphs. In particular, a general overview is firstly provided followed by a focus on major findings on GC, CRC, and HCC. Finally, a pathological and a molecular perspective are provided.

## 3. Immunotherapeutic Strategies: A General Overview

Activation of immune system against cancer might derive from active immunotherapeutic strategies, such as the adoption of cytokines, cancer vaccines, and immune checkpoints inhibitors or from a passive immunization mediated by adoptive cellular therapy (ACT) or monoclonal antibodies [14, 36]

The first attempts of active host immunity stimulation were based on the adoption of cytokines, in particular interferon-*γ* (IFN-*γ*), interleukin-2 (IL-2), IL-10, or GM-CSF. However, cytokines-based strategies are not adopted in clinical practice since results of trials are dated and inconclusive [37].

Cancer vaccines, as active immunotherapy, were firstly investigated 30 years ago. They are designed to activate and expand tumour-specific T cells with the potential to produce a persistent or even permanent anticancer effect. The ideal vaccine is easy to administer, offers prolonged protection, and induces relatively low toxicity. Although many trials investigated the possible role of peptide, protein, whole tumour cells, or DC-based vaccines in GI cancers [37], to date none entered the clinical practice. However, a renewed enthusiasm derived from a new class of recombinant immunogenic protein fused with a novel cell-penetrating peptide (Z12). This compound is able to promote efficient protein loading into the antigen processing machinery of DC and to lead to multiepitopic MHC class I and II restricted presentation. This novel vaccine elicited an integrated and multiepitopic immune response with persistent CD8+ and CD4+ stimulation in different tumour models [38] and will be soon investigated in human GI models.

Undoubtedly, immune checkpoints inhibitors are the real game changers. The anti-CTLA-4 ipilimumab and the anti-PD-1 mAbs, pembrolizumab and nivolumab, were firstly approved by the US FDA for the treatment of metastatic melanoma in 2011 and 2014, respectively [39, 40]. Several trials investigated or are currently investigating such mAbs in GI cancer with very promising preliminary results. As an example, pembrolizumab received the breakthrough therapy designation in mCRC cancers with microsatellite instability in November 2015 [41]. Despite some practice changing results have been obtained, many efforts are currently made in order to identify subgroup of patients benefitting from these agents and to design newer strategies involving the association of standard treatment with immunotherapy. Moreover, new molecules are under investigation such as the anti-PD-L1 avelumab and atezolizumab in GC and the anti-CTLA-4 mAb tremelimumab in HCC patients.

Among passive immunization strategies, adoptive cell therapy (ACT) is based on the passive transfer of tumour-specific T cells into a tumour-bearing host for the direct destruction of tumours. Briefly, T cells are collected from the tumour, draining lymph nodes or peripheral blood, and are activated and expanded in vitro. The first clinical trial of ACT in advanced cancers adopted lymphocytes-activated killer (LAK) cells. Since then, the innovative ACT with tumour-infiltrating immune cells (TILs) has been developed taking advantage of lymphocytes with demonstrated ability to recognise the tumour. ACT with TILs isolated from resected tumours, expanded ex vivo, and administered to patients in combination with IL-2 has demonstrated a 50% response rate in patients with metastatic melanoma [42, 43]. Since TILs have been isolated from a variety of GI cancers, this approach is currently under investigation in the metastatic setting [44]. The most recent ACT treatment adopts engineered T cells able to express chimeric antigen receptors (CARs) specific for CEA. CARs engage their target independently from antigen processing process and from MHC. Thus, CAR therapy is advantageous when MHC class I is downregulated [45]. Since T cells are ubiquitously expressed, targeting self-antigens might cause serious immune-related toxicities and safety concerns are still unsolved [45].

Finally, monoclonal antibodies (mAbs) commonly adopted in GI cancers represent the most relevant example of passive immunotherapy strategy. However, a wide body of literature is already available regarding this topic and it is not discussed in the present review.

## 4. Focus on Gastric, Esophageal, and Pancreatic Cancers

The first promising data about immunotherapy in GC or gastroesophageal junction cancer (GEJC) came from anti-PD-1 agents. The phase Ib study KEYNOTE-012 was designed to assess the safety and activity of pembrolizumab in GC and GEJC and the predictive role of PD-L1 expression in those malignancies. Primary endpoints were safety and response rate (RR). Toxicity profile was manageable; among 36 evaluable patients, RR was 22% (95% CI 10–39). No association between PD-L1 expression and clinical responses to pembrolizumab was observed [46]. Moving from the promising KEYNOTE-012 results, two trials are currently ongoing: the KEYNOTE 061 is evaluating pembrolizumab versus paclitaxel after progression to a first-line platinum-based therapy [47] and the KEYNOTE 062 is randomizing patients to receive pembrolizumab as monotherapy or platinum and 5-FU in association with pembrolizumab or placebo in the first-line setting [48] (see Table 3).

Pembrolizumab as single agent was also tested in esophageal cancer (EC) in the multicohort, phase Ib KEYNOTE-028 trial. In this study, 23 pretreated patients with either squamous cell carcinoma (SCC) or adenocarcinoma of the esophagus or GEJC were treated. Encouraging results were reported: the ORR was 30.4% and 52.2% in SCC and adenocarcinoma patients, respectively. Six- and 12-month progression free (PF) rates were 30.4 and 21.7%, respectively [49].

In the randomized phase III trial ONO-4538/BMS-936558, the anti-PD-1 nivolumab was tested as monotherapy versus placebo in advanced GC and GEJC after second or later lines. This study met all its endpoints. In detail, mOS was 5.3 versus 4.1 months (HR = 0.63, 95% CI 0.50–0.78, *p* < 0,0001) and mPFS was 1.61 versus 1.45 months (HR = 0.60, 95% CI 0.49–0.75, *p* < 0,0001) in the nivolumab (*N* = 330) and in the placebo arm (*N* = 163), respectively [50]. The shape of the curve shows that only a subgroup of patients derives benefit from the treatment reaching a long lasting disease control and response to treatment.

Nivolumab has also been tested in 65 patients affected by advanced esophageal SCC in a Japanese single-arm phase II trial. Patients received one or more previous treatment and were not preselected by PD-L1 status. The preliminary results showed durable activity with a manageable safety profile, with median OS of 12.1 months in 64 evaluable patients [51].

Another promising immunotherapy agent is the anti-PD-L1 avelumab, which has been tested in patients with GC or GEJC in the phase Ib trial JAVELIN. Patients were eligible if treated with a first-line chemotherapy based regimen and grouped by progression status after first line: patients achieving disease control during first line received avelumab as switch maintenance (*N* = 89) and those with progressive disease after chemotherapy received it as second line (*N* = 62). Primary endpoint was safety. An acceptable safety profile was shown. ORR was 9.0% and 9.7% in the 2 subgroups, respectively [52]. Given the promising results of this trial, JAVELIN Gastric 100 and JAVELIN Gastric 300 phase III trials are now ongoing [53, 54].

Less encouraging results come from anti-CTLA-4 agents, which showed higher toxicity and lower efficacy than anti-PD-1 in gastric and esophageal malignancies. The reasons for these differences are still debated. No objective responses were observed with the anti-CTLA-4 tremelimumab, tested as second-line treatment in a phase II trial in advanced GC and EC [55]. Similarly, ipilimumab was compared to best supportive care (BSC) in a randomized phase II trial, in pretreated patients with metastatic or locally advanced GC or GEJC and survival parameters were similar between the two arms [56].

In order to enhance the activity of anti-CTLA-4 antibodies, combination treatments with anti-PD-1 have been tested. The checkMate-032 is a phase I/II multicohort trial that randomized 160 pretreated patients to receive (1) nivolumab alone 3 mg/kg, (2) nivolumab 3 mg/kg plus ipilimumab 1 mg/kg, or (3) nivolumab 1 mg/kg plus ipilimumab 3 mg/kg. A notable RR was seen in each arm, with an overall DCR of 38%. Of interest, the ORR in patients with PD-L1-positive (≥1%) and PD-L1-negative (<1%) tumours was 27% and 12%, respectively, suggesting that PD-L1 expression may increase response rates. The highest ORR (26%) and mOS (6.9 months) were observed in arm 3 (nivolumab 1 mg/kg and ipilimumab 3 mg/kg) [57]. Given these interesting findings, the phase III trial CheckMate-649 investigating nivolumab plus ipilimumab versus FOLFOX/XELOX in untreated patients is ongoing.  Table 1 shows ongoing trials in this setting.

Pancreatic cancer models have been widely adopted in order to identify the immunotherapeutic rationale in GI cancer; however, data derived from early phase clinical trials yielded no benefit in pancreatic cancers. In particular, negative results derived from checkpoint inhibitor and vaccination trials [58]. Future clinical trials will test combination approaches in order to overcome immunosuppressive intratumour mechanisms and/or to increase the immunogenicity of microenvironment.

## 5. Focus on Colorectal Cancer

The first data on immunotherapy in CRC came from 1981, when the role of vaccines as immunotherapy was explored, based on the rationale of activating host defense against tumour-specific or tumour-associated antigens by means of the injection of autologous tumour cells with an immunomodulator (Bacillus Calmette-Guérin (BCG)). Preclinical models showed that the injection of BCG and tumour cells (OncoVAX®) was able to activate systemic immunity and stop the tumour burden [59].

The efficacy of OncoVAX was subsequently evaluated in the adjuvant setting in three phase III clinical trials, where patients were randomized to receive surgical resection only or surgical resection plus vaccination. The first study (8102) was initiated in 1981 and enrolled 98 patients with stages II and III CRC. The primary endpoints, OS and disease-free survival (DFS), were not reached (HR for OS = 1.75, *p* = 0.68; HR for DFS = 1.58, *p* = 0.147). However, in the subgroup analyses a significant benefit of OncoVAX was seen in patients with colon cancer (HR for OS = 2.83, *p* = 0.02; HR for DFS = 2.67, *p* = 0.039) and not in those with rectal cancer (HR for OS = 1.13, *p* = 0.772; HR for DFS = 1.05, *p* = 0.905) [60]. The phase III 5283 trial enrolled 412 colon cancer patients with stages II and III; no differences in OS and DFS were observed [61]. Lastly, in the phase 8701 III trial, 254 patients with stages II and III colon cancer patients were enrolled; the vaccine was centrally manufactured and was administered 4 times instead of 3. A 44% risk reduction for disease recurrence was observed in patients treated with OncoVAX (*p* = 0.023). In the subgroup analyses, the efficacy was only observed in stage II patients (61% risk reduction for disease recurrence) [62].

A meta-analysis including the 3 above reported trials showed an improvement in recurrence-free interval by OncoVAX with an annual odds reduction of 25 ± 13% (*p* = 0.05). The subgroup analysis by stage showed a predominant improvement in stage II patients (*p* = 0.05) [61]. According to these promising data, a multicenter phase III trial with OncoVAX in stage II patient is currently ongoing (Table 1).

Virus modified vaccines were also investigated in CRC. In particular, the Newcastle disease virus-infected (NDV) autologous modified vaccine, obtained admixing a nononcolytic strain Ulster of NDV with irradiated autologous tumour cells was tested in patients undergoing radical liver resection [63]. The results of a randomized phase III trial with NDV autologous modified vaccine in patients who undergone radical resection of CRC liver metastases were published at the end of 2000s. In this study, 51 patients were enrolled. No differences in OS (primary endpoint) and in DFS (secondary endpoint) were detected. However, in the subgroup analyses, a significant advantage was observed in patients with colon cancer with respect to OS (HR 3.3,   *p* = 0.042) and DFS (HR 2.7,   *p* = 0.047) but not in those with rectal cancer [64].

Data emerging from cancer vaccines have not yet altered the clinical practice. Several trials are currently ongoing in adjuvant and in metastatic settings with the aim of improving vaccines immunogenicity and of identifying a subset of patients amenable of this kind of treatments.

Conversely, the striking results obtained from immune checkpoint inhibitors trials lead to the introduction of new therapeutic options in mCRC. The first data regarding initial success of immune checkpoint inhibitors in mCRC were presented in mid-2015, when the results of the phase II KEYNOTE 016 trial with pembrolizumab in patients with refractory metastatic tumours were published. In this study, three cohorts of patients were recruited: (1) cohort A: patient with high microsatellite instability (MSI-H) or deficient mismatch repair (dMMR) mCRC (*n* = 11); (2) cohort B: patients with microsatellite stability (MSS) or proficient (p)MMR mCRC (*n* = 21); and (3) cohort C: patients with MSI-H non-mCRC cancers (*n* = 9). Immune-related objective response (iORR) rates were 40%, 0%, and 71% in the 3 groups, respectively; the median PFS and OS were not reached in cohort A; 2.2 and 5.0 months, respectively, in cohort B (HR for PFS = 0.10, *p* < 0.001, HR for OS = 0.22, *p* = 0.05) [41]. For the first time the activity of an anti-PD1 was demonstrated in patients with MSI-H while no effect was observed in MSS mCRC patients. As possible explanation of such results, it was demonstrated that tumours with MSI-H are characterized by a high burden of somatic mutations that can be recognised by the patient's immune system. As a supplementary proof, MSI-H tumours were found to be characterized by a dense immune infiltration and a cytokine-rich environment [65].

Based on these results, on November 2, 2015, the FDA granted “breakthrough therapy designation” for pembrolizumab in advanced CRCs with high microsatellite instability (MSI-H). To further explore this strategy, the KEYNOTE 164 trial was planned [66]; in this trial, pretreated MSI-H mCRC patients are candidate to receive pembrolizumab 200 mg every 3 weeks. Moreover, a phase 3 study of pembrolizumab versus investigator choice chemotherapy for MSI-H mCRC in first line is ongoing (KEYNOTE 177) [67].

One year later, at the 2016 ASCO Annual Meeting, several encouraging preliminary data on immune-checkpoint inhibitors in the treatment of mCRC were presented, including the update of the KEYNOTE 016 trial [68], a new treatment strategy adopting a combination of anti-CTLA4 and anti PD1 (the CHECKMATE 142 trial) [69], and a phase Ib study combining a MEK inhibitor and an anti-PD-L1 in patients with microsatellite stable (MSS) tumours [70].

The phase II CHECKMATE 142 trial investigates nivolumab plus or minus ipilimumab in patients with MSS and MSI-H mCRC patients in advanced lines of treatment. In the MSI-H cohort, ORR was 25,5% in patients receiving nivolumab (*N* = 47) and 33,3% in those receiving ipilimumab plus nivolumab (*N* = 27). Data presented at ASCO GI 2017 on 72 patients treated with nivolumab showed encouraging results for ORR, 12-month PF rate, and 12-month survival rate (31%; 48,4%; and 73.8%, resp.). Responses were observed regardless of tumour or immune cell PD-L1 expression,* BRAF*,* KRAS* mutation status, or clinical history of Lynch syndrome. Centrally revised data identified 2 patients experiencing complete response. This data represents a big step forward in the treatment of advanced mCRC and we perfectly agree with the conclusion of the authors stating that nivolumab should be considered a new standard of care for patients with previously treated MSI-H advanced CRC [70]. A new cohort of the trial is evaluating the activity of nivolumab and ipilimumab as first-line treatment (Table 1).

Data presented so far are highly significant in the subgroup of MSI-H patients while results in MSS cases are disappointing. RRs in patients with MSS treated with nivolumab or ipilimumab plus nivolumab were 10% and 0%, respectively, with overall poor PFS and OS [70]. Thus, many efforts are ongoing in order to identify possible immunotherapeutic strategies in MSS. In preclinical models, MEK inhibition alone increased the tumour-infiltrating CD8^+^ T cells and induced MHC-I upregulation, the combination of MEK inhibition with an anti-PD-L1 resulted in synergistic and durable tumour regression [71]. In a cohort of 23 mCRC patients receiving the MEK inhibitor cobimetinib and the anti-PD-L1 antibody atezolizumab, the ORR was 17% with 4 partial responses and 5 disease stabilizations. Among responders 3 out of 4 were MSS, thus leading to hypothesizing a possible effect for such strategy in this group of patients. A phase III trial is currently investigating atezolizumab and cobimetinib versus regorafenib in refractory mCRC [71, 72]. Other association strategies of checkpoints inhibitors with chemotherapy or anti-VEGF are also under investigation in mCRC patients irrespective of MSI status. All immune strategies under investigation in CRC are summarized in Table 2.

## 6. Focus on Hepatocellular Carcinoma

Over the last decades, recombinant human interferon-alfa (IFN-*α*) has been extensively studied in patients with HCC, due to its previous use as immune-stimulatory antiviral agent. Both adjuvant and advanced settings have been investigated [73, 74].

Given the association of a specific HCC-directed immune response with a prognosis improvement, various targets among tumour associated antigens (TAA) or neoantigens have been investigated. Peptide based, DNA/RNA based, and DCs based vaccines have been tested in clinical setting, but efficacy data have been to date disappointing [75, 76]. In a phase 1 study published in 2012, two glypican-3 (GPC3) derived peptides restricted for HLA-A phenotypes induced specific CD8+ cells tumour infiltration; a peptide-specific cytotoxic T response was associated with longer OS, but only one out of 33 treated patients reached an objective response [77]. The same treatment was investigated in a more recent phase II single-arm study in the adjuvant setting after resection or RFA: among 41 patients evaluable, 31 (75,6%) experienced a recurrence, with a mOS of 20.1 months [78]. Telomerase-derived peptide [79], DNA, RNA, and DCs based vaccines have also been studied with overall negative results [80–86]. A new phase I clinical trial to evaluate the safety of an allogenic dendritic cell vaccine-COMBIG-DC in HCC patients is now recruiting participants (NCT01974661).

As for other GI cancers, most exciting results derived from data on immune checkpoint inhibitors. The first report came in 2013 from a pilot phase II study of tremelimumab, an anti-CTLA-4 mAb, in 20 patients with HCC and chronic HCV. Efficacy data showed a good antitumour activity, with a 17.6% RR, 76.4% DCR, and a median PFS of 6.5 months [87]; these results are remarkable when considering that the majority of patients were pretreated with sorafenib and had a Barcelona Clinic Liver Cancer (BCLC) stage C and 43% had a Child-Pugh stage B.

Several phase II/III trials with anti-PD-1/PDL-1 agents, alone or in combination with other compounds, are ongoing, with some preliminary data already reported. The most robust data presented so far are about the anti-PD-1 nivolumab, investigated alone or in combination with ipilimumab in a multicohort phase I/II study opened in 2012 for advanced HCC patients (CHECKMATE040). Four out of 5 scheduled cohorts have completed enrollment for a total of 576 HCC patients treated. Among 262 patients treated with nivolumab monotherapy, across dose escalation (*n* = 48) and dose expansion cohorts (*n* = 214), [88] a 20% RR was observed irrespective of dose, HCV, or HBV infection status and PDL-1 expression on tumour cells. Median duration of response was 9.9 months; median OS was 15.0 months and 13.2 months in dose escalation and expansion cohort, respectively. These data are very promising, especially considering the overall poor prognosis of HCC patients. In the Sorafenib Hepatocellular Carcinoma Assessment Randomised Protocol (SHARP) RR, survival rate at 12 months was 2% and 44%, respectively, and mOS was 10.7 months [89, 90]. Thus, a phase III study of nivolumab versus sorafenib in treatment-naïve patients has been planned and is already ongoing (CHECKMATE 459) [91].

The anti-PDL-1 durvalumab demonstrated clinical activity in several solid tumours, including 19 HCC patients, in a phase 1 study published in 2014 [92]; a randomized open-label phase II study is currently ongoing with durvalumab, tremelimumab, or the combination of the two compounds in patients with unresectable HCC. Pembrolizumab is also under investigation in HCC: a phase II open-label study just completed the enrollment with sorafenib intolerant or progressed patients (Keynote 224), and a phase III study planning to enroll 408 second-line patients is recruiting patients (Keynote 240). Ongoing studies are summarized in Table 3.

## 7. Possible Biomarkers for Immunotherapy: The Pathologist Perspective

The introduction of immunotherapies as possible treatment options in GI cancers made the assessment of MSI status (especially for CRC) and PD-L1 expression crucial in the pathologic assessment of GI cancers. Overall, an adequate characterization of the immune microenvironment in cancer samples emerged as the driver diagnostic element for the identification of patients likely to benefit from specific immunotherapies [93, 94].

Among the others, the greatest focus has been on PD-L1 expression [95]. Increasing evidences pointed out to the association between PD-L1 and a higher burden of disease, more extensive metastatic involvement of lymph nodes, and poorer survival, in both esophageal and gastric cancers. Although PD-L1 testing by immunohistochemistry has been associated with a significant enrichment for populations with clinical benefit to anti-PD1 or anti-PDL1 therapies, no conclusive date have been reported so far [46, 57] and several factors are limiting its use in the clinical practice. Above all, different threshold levels have been adopted for the identification of positive samples in different tumour types [96]. Several reports pinpointed the predictive value of PD-L1 expression on infiltrating immune cells instead of tumour cells [97]. Most companies have developed their own companion PD-L1 immunohistochemistry diagnostic assay characterized by different antibody-specific features. Of course, this diversified request for immunohistochemical testing and the related need of antibody/company-specific immunostainers is inconsistent with the current practice of most surgical pathology laboratories.

From a general perspective, the identification of consistent biomarkers to be introduced into clinical practice is affected by (i) the inherent biological heterogeneity of tumour microenvironment; (ii) the complexity of novel immunotherapeutic regimens and the combination of immunotherapy with other target therapeutics; (iii) the variability on molecular biology testing; (iv) the inconsistent aptitude of formalin-fixed paraffin-embedded (FFPE) preparations with many downstream molecular biology techniques [98]; (v) the significant discrepancies in the proposed biomarker evaluation systems [99]; (vi) the need of integrated diagnostics (i.e., histology, immunophenotyping, and molecular profiling), not always available in “spoke” surgical pathology units.

Similar considerations might be drawn for MSI status assessment that represents the only well-established predictive biomarker for immunotherapy response in mCRC. MSI status is assessed by means of immunohistochemistry evaluating altered expression of mismatch repair proteins (i.e*.,* MLH1, PMS2, MSH2, and MSH6) or by means of PCR techniques detecting mutations on BAT25, BAT26, D2S123, D5S346, and D17D250, according to the Bethesda panel guidelines [100].

Due to the association of MSI status with a higher mutational and neoantigen burden, also more sophisticated next-generation sequencing approaches have been successfully applied in the evaluation of mutational load in immunotherapy clinical trials, and these methods allow the identification of other hypermutated tumour classes such as those characterized by dysfunctions in DNA polymerases (POLE) [101]. However, even these promising data, neither mutational load analysis nor the evaluation of the mismatch repair machinery status, have been included in the clinical selection of the patients undergoing immunotherapy, so far.

Both PD-L1 expression and MSI might play a role as positive predictive factors for GC and immunotherapy according to the data from the Cancer Genome Atlas (TCGA) Research Network project [22].

The presence of tumour-infiltrating lymphocytes (TILs) is an indirect sign of disease control through immune mechanisms and has been evaluated as a predictive biomarker for checkpoint inhibitor immunotherapy [102, 103]. Beside these therapeutic implications, the landmark studies of Jérôme Galon identified the prognostic value of the global assessment of the immune infiltrate (also known as immunoscore) in colorectal cancer and in other solid tumours [104, 105]. However, infiltrating lymphocytes evaluation still lacks intralaboratory and intrapathologist standardization and is not yet a widespread practice among the pathologists' community.

Because of all these challenging problems in the definition of ultimate optimized model for predicting tumour response to anti-PD1 or anti-PD-L1-based therapies, more technically complex combined biomarker strategies and/or comprehensive immune gene signatures have been also successfully tested. The limited amount of analysable material in preneoadjuvant biopsy specimens and the use of FFPE samples are, however, currently affecting the improvement of these approaches in the clinical setting.

Overall these data underline that an adequate personalized immunotherapy will be obtained only with the integration of traditional microscope-based biomarkers (such as the immunoscore) to more advanced FFPE-compatible genetic, genomic, and expression profiling strategies. A new revolutionizing era of diagnostic surgical pathology has started.

## 8. Back to the Bench: Biomarkers and Genetic Signatures as Predictive Factors

As stated above, traditional microscope-based techniques are not adequate to comprehensively assess the intriguing landscape of tumour benefitting from immunotherapy. Genetic signatures might be useful tools to identify predictive biomarkers able to help patients' selection.

Among proposed biomarkers, MSI-H status represents the only validated positive predictive factor for immunotherapy response in mCRC; however, it occurs in only 6% of patients. Given the high benefit deriving from immune checkpoint inhibitors in this setting in terms of OS, responses, and symptoms relief, recently, several papers have pointed out the importance of using gene expression profile to better identify those tumours that behave as MSI-H.

Tian et al. [106] adopted full genome expression data of stage II and stage III CRC to identify genes that correlate with MSI status. An MSI gene signature was developed and further validated in other external data set with an overall accuracy of about 90.6%. The strength of the MSI-signature is that it can identify the true MSI-H patients as well as a group of patients that are not MSI by conventional clinical tests but they are by signature. Those patients are defined as MSI-like and share the hypermutated status as pure MSI-H patients. Furthermore, they seem to not respond to 5-FU regimen as stage II MSI-H patients. If MSI-H mCRC patients benefit from immunotherapy, we can assume that also MSI-like patients will. Indeed, this is the rationale of one of the trials that will soon be run in the frame of the MoTriColor consortium (http://www.motricolor.eu/).

In line with these findings, more recently, Mlecnic et al. [107] performed a comprehensive analysis of the tumour microenvironment, immune gene expression, and mutational status in CRC, so-called immunoscore. They identified a high number of genes upregulated in MSI tumours (high immunoscore) versus MSS (low immunoscore). These genes were mainly associated with INF*γ* signalling, Th1 related cytokines, antigen presentation pathways, chemokine receptors, and chemokine and leucocyte migration. However, a high immunoscore was identified also in a subset of MSS tumours and was not observed in a certain number of MSI tumours. Both MSI-H and high immunoscore predicted favorable prognosis among CRC patients; however, data derived from multivariate analyses identified the immunoscore as a stronger predictor of good CRC patients' survival than MSI and proposed it as a stronger predictor of immunotherapy response than MSI.

Although those two studies do not question the role of MSI status in CRC in terms of increased immune infiltrates, higher frequencies of frame shift mutations, and favorable outcome, still they demonstrate that the canonical MSI tests may be not sufficient to fish out all the patients that have a common “MSI phenotype” and could benefit from immunotherapy. Interestingly enough, Zhao et al. [108] also identified a MSI-H mutation signature by using whole genome and whole exome sequencing. They confirmed this signature to be similar to germline DNA, thus meaning that a fraction of genetic variations arises through mutations escaping MSI. Most importantly, they identified a large number of recurrent indels that can be used to detect MSI and that are currently under implementation for clinical application. Moreover, they found that recurrent indels are enriched for the double-strand break repair (DBS) by homologous recombination (HR) pathway. All in all, these data indicate that the MMR pathway is not yet completely known and that in the future new biomarkers belonging to this pathway will need to be validated and used as predictive of response to immunotherapy. Moreover, the importance of those gene signatures has been shown only in CRC. Thus, further studies will be required to know if this applies to other tissues types that show microsatellite instability, such as gastric cancer, genitourinary tract malignancies, and esophageal cancer. Since MSI-H tumours are not that frequent especially in the metastatic setting, the use of gene expression profile could help in enlarging the group of patients who will benefit from such treatment. At the same time, this will also avoid that useless toxicity will be given to patients who seem to carry an MSI-H tumours but that by gene expression it is not defined as MSI-like or immunoscore positive or positive for the MMR-deficient mutation signature. A proof that a response to immunotherapy can also be observed in MSS tumours is provided by the case report published by Chen et al. [109]. Authors report indeed the case of a 64-year-old man who received pembrolizumab as second-line treatment for a HER2 positive metastatic gastric cancer. Clinical tests reported the tumour to be MSS and Epstein Barr negative and to not carry any mutations in the POLE gene, thus meaning carrying all the biomarkers that so far have been identified as negative predictors of response to immunotherapy. Although the authors did not investigate other biomarkers to understand why the patient responded to immunotherapy, based on the data here summarized, we can hypothesize that MSI clinical tests are not sensitive enough and that the integration of multiple tumour and immune response parameters such as protein expression, genomics, and transcriptomics may be necessary for accurate prediction of clinical benefit.

Finally, response to immunotherapy might not only be driven by the “genetic makeup” of the tumour and the way how the immune system reacts to it, but also by its regulation via other mechanisms such as the gut microbiota. Indeed two recent papers show its role in modulating the anticancer activity of CTLA4 and PD1 blockade. Vétizou et al. [110] elegantly showed that the gut microbiota can itself reduce the tumour volume and when combined with immunotherapy it further reduces the tumour size. This effect is driven only by certain microbiota composition, like the Bacteroides spp., which seem to be also regulated by ipilimumab itself. Life style and immunotherapy could change the gut microbiota. This in turn affects interleukin 12 dependent Th1 immune response which facilitates tumour control both in mice and in patients while sparing intestinal integrity. Moreover, the oral administration of* Bifidobacterium* associates with tumour effect and when combined with anti-PDL1 therapy nearly abolishes tumour outgrowth. Whether the role of the microbiota in modulating the response to immunotherapy is tissue specific is not yet clear. Thus, further investigation is required. If those data will be confirmed, we might consider in the future the use of stool to identify biomarkers and fecal transplantation to modulate the immune response.

## 9. Future Perspectives

Recently, ASCO proclaimed immunotherapy against cancer as “the advance of the year.” In particular, immune checkpoint blockade was heralded as a major breakthrough in cancer therapy in the last years [111]. However, there are still many challenges that must be overcome; in particular, in GI cancer many drugs are still in the early phase of development.

First of all, biomarkers identification and validation represent a major issue as discussed in the last 2 paragraphs of the review.

Secondly, clinicians still need to learn how to deal with response assessment in patients receiving immunotherapy. Conventional and nonconventional responses have been reported. As an example, patients experiencing a rapid disease progression need to be carefully evaluated with an expert radiologist, to exclude the occurrence of a pseudoprogression, identified as the burning of an inflammatory response that can simulate the onset of new lesions. Moreover, we have to be aware that a RECIST response might be observed after more than three months of treatment but can be persistent after occurrence [112]. It has also been proposed that RECIST criteria might not be adequate to assess immune response; although immune-related response criteria have been developed [112], they are still not universally adopted especially in clinical trials on GI immunotherapy [112].

Toxicity profile of immunotherapeutic agents represents another thorny issue. GI oncologists involved in clinical trials are facing a different adverse event scenario compared to the traditional chemotherapy one and need to improve their knowledge and skills to treat immune-related toxicities in a subset of patients who may already have baseline GI, liver function, and endocrine abnormalities from their underlying cancer or as complications from prior treatments. [113].

Finally the most efforts are focusing on the development of novel approaches to enhance this innovative strategy. All ongoing trials are shown in Tables 1–3. Promising trials have been evaluating innovative combination treatments (so-called “combo-immunotherapy”), that is, PD-1 or PD-L1 blockade in combination with (1) anti-CTLA4, (2) adaptive immunotherapy such as anti-LAG3, (3) innate immunotherapy such as TLRs agonists, (4) chemo- or radiotherapy, (5) drugs able to increase antigen presentation such as the COX-2, JAK1/2 inhibitor or the MEK inhibitor cobimetinib, and (6) targeted therapy (anti-HER2, anti-VEGFR2) [69, 114, 115].

From a clinician perspective, the use of immunotherapies in recent clinical trials gave us the opportunity to contribute to a paradigmatic shift in the treatment of GI cancers. We are glad to observe highly pretreated patients experiencing a dramatic clinical benefit after treatment start, with symptoms relief, long lasting disease stabilization, and an overall manageable safety profile. We are really feeling a revolution in the daily life of our patients. Every day we ask questions about future availability of clinical trials involving immunotherapeutic agents for GI cancers from our new and historical patients. We strongly believe that further steps of drugs development such as larger phases II and III clinical trials are warranted in order to answer unsolved question and to establish the efficacy of immunotherapeutic agents. A wide international involvement of experienced centers in the next clinical trials will break a potential unequal distribution of immunotherapeutic resources.

## Figures and Tables

**Table 1:** Ongoing studies on gastric, gastroesophageal junction, and esophageal cancers.

NCT identifier	Setting	Phase	Study interventions	Number of patients	Primary endpoint
*Checkpoint inhibitors*					
NCT02689284	Metastatic HER2+ GC/GEJC	Ib/II	Margetuximab+ pembrolizumab	52	MTD and MAD for margetuximab; duration of response; 12-month ORR
NCT02563548	Metastatic GC after 1st line	Ib	PEGPH20 +pembrolizumab	81	DLT; 18-month ORR
NCT02443324	Metastatic GC/GEJC and other tumours	I	Ramucirumab + pembrolizumab	155	DLT
NCT02589496	Metastatic GC/GEJC after first line	II	Pembrolizumab	40	2-year RR
NCT02901301	First-line HER2 + GC	Ib/II	Pembrolizumab + trastuzumab + capecitabine + cisplatin	49	RP2D; 6-week ORR
NCT02954536	First-line HER2+ GC/GEJC/EC	II	Pembrolizumab + trastuzumab + capecitabine + cisplatin	37	6-month PFS
NCT02318901	Unresectable HER2 + GC/GEJC	II	Pembrolizumab + ado-trastuzumab emtansine	90	RP2D
NCT02559687	EC (adenocarcinoma or squamous cell)/GEJC after 2nd line	II	Pembrolizumab	100	2-year ORR
NCT02494583	First-line GC/GEJC	III (random)	Pembrolizumab versus pembrolizumab + cisplatin + 5-fluorouracil or capecitabine versus placebo + cisplatin + 5-FU or capecitabine	750	44-month PFS and OS
NCT02370498	Second-line GC/GEJC	III (random)	Pembrolizumab versus paclitaxel	720	PFS, OS
NCT02564263	EC (adenocarcinoma or squamous cell) /GEJC after 1st line	III (random)	Pembrolizumab versus investigator's choice of standard therapy (paclitaxel, docetaxel, or irinotecan)	600	3-year PFS and OS
NCT02872116	Unresectable GC/GEJC	III (random)	Nivolumab + ipilimumab versus nivolumab + oxaliplatin + fluoropyrimidine versus oxaliplatin + fluoropyrimidine	1266	40-month OS in patients PD-L1 +
NCT02864381	Metastatic GC/GEJC	II (random)	GS-5745 + nivolumab versus nivolumab alone	120	2-year ORR
NCT02340975	Pretreated metastatic/GC/GEJC	Ib/II (random)	MEDI4736 + tremelimumab versus MEDI4736 versus tremelimumab	135	Phase Ib: DTL, Phase II: ORR and 6-month PFS
NCT02625623	3rd-line GC/GEJC	III (random)	Avelumab+ BSC versus chemotherapy (paclitaxel or irinotecan)+BSC or BSC alone	330	2-year OS
NCT02625610	1st-line GC/GEJC	III (random)	Maintenance with avelumab versus continuation of 1st-line chemotherapy	666	3-year OS and PFS

*Immunotherapy + radiotherapy*					
NCT02642809	1st-line EC	I	Pembrolizumab + brachytherapy	15	Tolerability and toxicity
NCT02830594	Pretreated EC/GC(GEJC	II	Pembrolizumab + external beam palliative radiation therapy	14	Biomarkers
NCT02735239	Metastatic EC	I/II	Durvalumab + oxaliplatin/capecitabine	75	AE, DLT, laboratory evaluations
*Vaccines*					
NCT02276300	Metastatic HER 2 + GC	I	HER2-derived peptide vaccination	12	Safety and tolerability
NCT02317471	Stage III gastric cancer	I/II	Vaccination with autologous tumour derived heat shock protein gp96	45	DFS
NCT02795988	Metastatic HER 2 + GC/GEJC	Ib/II	IMU-131 HER2/Neu peptide vaccine+ cisplatin and either 5-FU or capecitabine chemotherapy	18	RP2D, AE
*Cytokines*					
NCT01691664	Locally advanced EC	NS (random)	Radiation therapy alone or with DC-CIK cellular therapy	40	DFS
NCT01691625	Locally advanced EC	NS (random)	Concurrent chemoradiation with or without DC-CIK	50	Quality of life
NCT02504229	Metastatic refractory GC	II (random)	Chemotherapy with or without DC-CIK	80	PFS
NCT01783951	Metastatic refractory GC	I/II	S-1 with or without DC-CIK	30	PFS

*CAR-T cells*					
NCT02713984	Metastatic refractory HER 2 + GC	I/II	Anti-HER2 CAR-T cells	60	Toxicity
NCT02725125	Metastatic refractory GC	I/II	EPCAM-targeted CAR-T cells	19	DCR
NCT02617134	Metastatic refractory MUC1+ GC	I/II	Anti-MUC1 CAR-T cells	20	Toxicity
NCT02349724	Metastatic refractory CEA+ GC	I	Anti-CEACAR-T cells	75	Toxicity
NCT02862028	Metastatic refractory EGFR+ GC	I/II	Anti-PD-1CAR-T cells	20	ORR, DCR, OS, PFS
NCT03013712	Metastatic refractory EpCAM+ GC/EC	I/II	Anti-EpCAMCAR-T cells	60	Toxicity

GC, gastric cancer; GEJC, gastroesophageal junction cancer; EC: esophageal cancer; NA, not assessed; MTD, maximum tolerated dose; MAD, maximum administered dose; DTL, dose limiting toxicity; ORR, overall response rate; OS, overall survival; PFS, progression-free survival; DFS, disease-free survival; NS, not specified; RP2D, recommended dose of phase II; AE, adverse events; DCR, disease control rate; BSC, best supportive care; DCR disease control rate.

**Table 2:** Ongoing studies on colorectal cancers.

NCT identifier	Setting	Phase	Study interventions	Number of patients	Primary endpoint
*Checkpoint inhibitors*					
NCT03026140	Early stage colon cancer	II	Nivolumab + ipilimumab versus nivolumab + ipilimumab + celecoxib	60	Safety
NCT02260440	Chemorefractory mCRC	II	Pembrolizumab + azacitidine	40	ORR
NCT02997228	MSI-H mCRC	III	mFOLFOX6 bevacizumab versus atezolizumab versus atezolizumab + mFOLFOX6 bevacizumab	439	PFS
NCT02870920	Chemorefractory mCRC	II	BSC + durvalumab + tremelimumab versus BSC	180	OS
NCT02788279	Chemorefractory mCRC	III	Atezolizumab versus cobimetinib + atezolizumab versus regorafenib	360	OS
NCT02991196	mCRC	I	DS-8273a + nivolumab	20	DLTs; MTD; ORR; DCR; TTP; PFS;
NCT02713373	Unresectable mCRC	I-II	Cetuximab + pembrolizumab	42	PFS; ORR; safety and tolerability
NCT02981524	mCRC	II	CY/GVAX + pembrolizumab	30	ORR
NCT02754856	mCRC with resectable CLM	I	Tremelimumab + MEDI4736 + FOLFOX bevacizumab	35	Feasibility
NCT02933944	RAS mutmCRC	I	TG02 versus TG02+ pembrolizumab	20	Safety; irORR
NCT02860546	MSS mCRC	II	TAS-102 + nivolumab	35	irORR
NCT02948348	Locally advanced RC	I-II	Chemoradiotherapy with capecitabine → nivolumab → surgical therapy	50	PCR
NCT02437071	mCRC	II	Pembrolizumab+ radiotherapy versus pembrolizumab+ ablation	48	ORR
NCT02060188 (CheckMate 142)	mCRC	II	Nivolumab/nivolumab + ipilimumab/nivolumab + ipilimumab 1st line/nivolumab + ipilimumab + cobimetinib/nivolumab + BMS-986016/nivolumab + daratumumab	260	ORR
NCT02512172	mCRC	I	Oral CC, 486 & MK-3475 versus romidepsin & MK-3475, versus oral CC, 486 & romidepsin & MK-3475	30	Degree of change in TIL
NCT02227667	mCRC	II	MEDI4736	48	ORR
NCT02563002 (KEYNOTE 177)	MSI-H mCRC	III	Pembrolizumab versus investigator's choice of standard of care	270	PFS

*Vaccines*					
NCT02448173	Stage II	III	OncoVAX + surgery versus surgery	550	DFS
NCT01890213	Stage III	I	AVX701	12	AE
NCT02718430	mCRC with CLM	I	VXM01	24	Safety and tolerability
NCT01741038	mCRC	II-III	AlloStim® + cryoablation versus AlloStim + physician's choice (PC)	450	OS
NCT02615574	Refractory mCRC	II	áDC1 vaccine+ CKM	44	OS

*Cytokines*					
NCT02415699	Stage III	II-III	DC-CIK + chemotherapy versus chemotherapy	100	DFS
NCT02280278	Stage III	III	Adjuvant CT → CIKCC versus adjuvant CT	550	DFS
NCT01929499	Stages II-III	II	Adjuvant CT + synchronous CIKCC versus adjuvant CT → CIKCC versus adjuvant CT	210	DFS
NCT02466906	Stage III	II	RhGM-CSF versus placebo	60	DFS
*Oncolytic virus*					
NCT01274624	KRAS	II	REOLYSIN® + FOLFIRI, bevacizumab	32	DLTs
NCT01622543	mCRC	II	FOLFOX + bevacizumab + reolysin versus FOLFOX + bevacizumab	109	PFS
*Adoptive cell therapy study*					
NCT03008499	mCRC	I-II	High-activity natural killer versus no special treatment	18	Relief degree of tumours evaluated by RECIST
NCT02577588	mCRC	I	Reactivated T cells	10	DLTs

*Adjuvant therapy*					
NCT01545141	Resectable CRC	I-II	Surgery versus chemokine modulatory regimen (a combination of IFN, celecoxib, and rintatolimod prior to surgery	50	Change in the number of tumour-infiltrating CD8+ cells

*Immune modulators therapy*					
NCT02077868 (IMPALA)	mCRC	III	Maintenance versus MGN1703	540	OS
NCT02413853 (PRIMIER)	mCRC	II	PRI-724 + mFOLFOX6/bevacizumab versus mFOLFOX6/bevacizumab	100	PFS

GC, gastric cancer; GEJC, gastroesophageal junction cancer; EC: esophageal cancer; NA, not assessed; MTD, maximum tolerated dose; MAD, maximum administered dose; DTL, dose limiting toxicity; ORR, overall response rate; OS, overall survival; PFS, progression-free survival; DFS, disease-free survival; NS, not specified; RP2D, recommended dose of phase II; AE, adverse events; DCR, disease control rate; BSC, best supportive care; DCR disease control rate.

**Table 3:** Ongoing studies on hepatocellular carcinoma.

Trial (*ClinicalTrials.gov* identifier)	Setting	Phase	Study interventions	Number of patients	Primary endpoint
*Checkpoint inhibitors*					
NCT02576509 CheckMate459	1st line	III	Nivolumab versus sorafenib in first line	726	OS, ORR
NCT02702414 Keynote 224	2nd line	II	Pembrolizumab in second line	100	ORR
NCT02702401 Keynote 240	2nd line	III	Pembrolizumab versus BSC in second line	408	PFS, OS
NCT01658878 CheckMate 040		I/II	Nivolumab versus nivolumab + Ipilimumab nivolumab	620	AEs, SAEs, ORR
NCT02519348	Advanced	II	Tremelimumab + MEDI4736 versus tremelimumab versus MEDI4736	144	AEs, SAEs, DLTs
*Vaccines*					
NCT01974661	Advanced	I	COMBIG-DC (allogeneic dendritic cells) cancer vaccine	18	Safety and tolerability
*Cytokines*					
NCT02632188	Resected	I/II	SURGERY → DC-PMAT treatment	60	PFS
NCT02873442	Advanced	I/II	TACE versus TACE + precision cell immunotherapy	40	PFS, OS
NCT02487017	Advanced	II	TACE versus TACE + DK-CIK		OS
*CAR-T cells*					
NCT02729493	Advanced	II	EPCAM-targeted CAR-T cells	25	DCR
